# Identification of cellular heterogeneity and immunogenicity of chondrocytes *via* single-cell RNA sequencing technique in human osteoarthritis

**DOI:** 10.3389/fphar.2022.1004766

**Published:** 2022-09-29

**Authors:** Xinyue Hu, Zhuang Li, Mingliang Ji, Yucheng Lin, Yuzhi Chen, Jun Lu

**Affiliations:** ^1^ School of Medicine, Southeast University, Nanjing, Jiangsu, China; ^2^ Department of Orthopaedic Surgery, Zhongda Hospital, School of Medicine, Southeast University, Nanjing, Jiangsu, China

**Keywords:** single-cell RNA sequencing (scRNA-seq), osteoarthritis, heterogeneity, immunogenicity, bulk RNA sequencing

## Abstract

**Background:** Osteoarthritis (OA) has placed a heavy burden to the economy and humanistics. To explore the biological functions and markers of chondrocytes contributes significantly to the accurate diagnosis and targeted treatment of OA.

**Methods:** We systematically analyzed the immunogenicity and biological function of varied chondrocytes at single cell resolution, and identified the chondrocyte subtypes and biomarkers involved in the development of OA, which are verified in the bulk sequencing cohort.

**Results:** Based on previous study, we defined eight subtypes of chondrocytes with different biological functions, finding out that effector chondrocytes (ECs) and fibrocartilage chondrocytes (FCs) may promote the development of OA. Compared with other chondrocytes, ECs and FCs show stronger immunogenicity. FCs mainly affects the degeneration of cartilage caused by fibrous degeneration, while ECs mainly exerts immune function and causes tissues inflammation. In addition, the canonical gene markers of EC and FC assist with the prediction of OA, which has been verified in Bulk RNA sequencing data from two GEO datasets.

**Conclusion:** In summary, this study provides a new perspective for the exploration of cellular heterogeneity and pathophysiology in OA and will make contribution to the accurate diagnosis and targeted treatment of OA.

## Introduction

Osteoarthritis (OA) is one of the most prevalent chronic joint diseases and a leading cause of progressive joint dysfunction (Edwards et al., 2015). On a global scale, OA has placed a heavy burden to the economy and humanistics and is expected to become the major cause of disability in patients aged over 40 years by 2040 (Thomas et al., 2014; Xie et al., 2016). The primary feature of OA is the impairment of articular cartilage homeostasis, which is followed by cartilage degradation and synovitis (Goldring and Otero, 2011; Jin et al., 2015; Wang and Chen, 2016). The articular cartilage is a physiologically non-self-renewing seavascular tissue composed of chondrocytes (Jiang and Tuan, 2015). Current evidence strongly supports that chondrocytes are involved in the pathology of OA owing to its specific phenotypes (Jiang and Tuan, 2015). However, there remains no chondrocytes-targeting prediction and treatment strategies for OA by now. In this context, it is essential to research the role of chondrocytes in immunoregulation and their pathophysiological changes during the progression of OA. Most of the existing studies were concentrating on the repair of cartilage tissues through stem cell transplantation (Johnson et al., 2012; Trounson and McDonald, 2015). Nevertheless, the results are limited due to the largely unknown information including the detailed subtypes of chondrocytes and their effects on OA, and the lack of biomarkers available to predict OA progression.

Chondrocytes are derived from the differentiation of condensed mesenchymal stem cells (MSCs) and then form articular cartilage, whose fibrocartilage matrix is composed of proteoglycan and collagen that are produced by chondrocytes (Chaly et al., 2015; Baboolal et al., 2016). In previous research, chondrocytes were subclassified by their developmental origin, site, morphology and molecular function according to experience. The first three subtypes are proliferative chondrocytes (ProCs), prehypertrophic chondrocytes (preHTCs) and hypertrophic chondrocytes (HTCs) (St-Jacques et al., 1999; Saito et al., 2010; Prein et al., 2016). ProCs are cells located to the proliferative zone; preHTCs can induce cell differentiation toward hypertrophy; and HTCs are capable of regulating the mineralization of cartilage matrix. Recently, two new subtypes of chondrocytes have been identified: senescent cells (SNCs) and cartilage progenitor cells (CPCs) (Koelling et al., 2009; Jiang and Tuan, 2015; Worthley et al., 2015; Childs et al., 2017; Jeon et al., 2017). SNCs feature senescence-associated secretory phenotype (SASP), which is beneficial for the development of OA (Jeon et al., 2017); CPCs express stem cell surface markers and can differentiate along multiple lineages, with the self-renewal capacity to maintain the repair of cartilage tissues (Koelling et al., 2009; Jiang and Tuan, 2015). While in most cases, such experience-based classification is not accurate, and there are limited markers which are available to recognize and differentiate these subtypes. As a consequence, it is difficult to carry out relevant studies. By the march of high-throughput sequencing technique, tissue heterogeneity has been studied at the molecular level and the sub-classification of chondrocytes in Human OA holds enormous promise for research.

The traditional Bulk RNA-sequencing technique determines the average expression of a gene in each cell at the tissue level, which fails to help study the cartilage tissue heterogeneity at the cellular level. The emergence of single-cell RNA sequencing (scRNA-seq) technique has compensated for the deficiency as it can provide transcriptome data of single cells. Ji et al. (2019) preliminarily made a profile showing the heterogeneity of chondrocytes in OA based on the scRNA-seq technique. Based on their research, we identified the sutypes of chondrocytes involved in OA progression and explored their immunogenicity via further bioinformatics analysis. In the meantime, we integrated the scRNA-seq data and the Bulk RNA-seq data from GEO to validate the performance of different cell subtypes and gene markers in diagnosis of OA. Findings of our study further reveal the biological properties of chondrocytes in OA and provide new ideas for the precision diagnosis and targeted therapy of OA.

## Materials and methods

### Data source

scRNA-seq matrix was obtained from the GSE104782 dataset uploaded by Ji et al. (2019) and the detailed information could be found in their study. Patient information was obtained from their Supplementary Material, including 1,600 chondrocytes samples from 10 OA patients. Severity score of each cell was calculated as described in their study (a lower severity score indicates a higher degree of disease severity).

Bulk RNA-seq data from two GEO datasets, GSE51588 (Chou et al., 2013) (GPL13497; OA, *n* = 40; Normal, *n* = 10) and GSE114007 (Fisch et al., 2018) (GPL18573; OA, *n* = 18; Normal, *n* = 18), were downloaded as validation data.

### Quality control, clustering analysis and sub-classification

The scRNA-seq data were processed for quality control, clustering and identification of marker genes using the R package Seurat (Version 3.0.1) (Butler et al., 2018). The PercentageFeatureSet function was used to examine mitochondrial genes, and cells with mitochondrial gene >5% and gene number >5,000 or <500 were excluded. Normalization was fulfilled using the NormalizeData and ScaleData functions of Seurat. PCA was performed to identify the main cell clusters with the appropriate PC number using the FindClusters function, which were visualized by 2D UMAP. Eventually, 1,343 cells were included for further analysis.

The FindAllMarkers function was applied to identify marker genes of each cell cluster with the “biomed” (Likelihood-ratio test), following logFC >0.25 and expression >25%. Chondrocyte type was identified according to the markers described by Ji et al. (2019) GO analysis (Chen et al., 2009) was performed using the ToppGene, and the GO terms with *p* < 0.05 were displayed. DO analysis (Yu et al., 2012) was conducted using the package “Clusterprofiler” with the significance threshold set as *p* < 0.05. Single-cell trajectory analysis was performed using the package “monocle” in marker genes (Trapnell et al., 2014) and the results were visualized in a 2D diagram.

### Copy number variation estimation

R package “inferCNV” was applied to estimate the copy number variation (CNV) of each cell type (Patel et al., 2014). We used all cell types as the reference background and set “denoised”. Finally, we use a threshold of 0.1 to detect CNV. For more details, please refer to the original article (Patel et al., 2014).

### Cell-cell communication analysis

Python package “CellphoneDB” (Vento-Tormo et al., 2018) was used to recognize the ligand-receptor interactions between cell clusters with a value of *p* < 0.01 set at significance. R package “Cellchat” was applied to infer the biological pathways involved in the cell-cell interactions (Jin et al., 2021).

### Functional enrichment analysis

ssGSEA was used to estimate the immune pathway activity of chondrocytes, with the background gene set obtained from the previous literature (Liang et al., 2020).

GSVA and GSEA were performed using the KEGG gene set downloaded from the MSigDB database (http://www.gsea-msigdb.org/gsea/msigdb/). The differential gene sets between cell clusters were explored using the R package “GSVA” with the default parameters.

GSEA was performed to analyze the enrichment of the gene sets with a value of *p* < 0.05 for cut-off.

### Bulk RNA-seq data validation

ssGSEA was performed to estimate the abundance of chondrocytes in the two independent datasets with Bulk RNA-seq data based on corresponding gene markers. PCA was conducted to evaluate the ability of cell clusters to differentiate OA tissues from normal tissues. ROC curve was generated to explore the diagnostic performance of the gene markers.

### TF-mRNA-miRNA regulatory network establishment

Upstream TF and miRNA of gene markers were predicted through the Enrichr (https://maayanlab.cloud/Enrichr/) database (*p* < 0.05). mRNA-TF pairs were predicted via TRANSFAC and JASPAR algorithms (Kuleshov et al., 2016), and miRNA-mRNA pairs were predicted through the mirTarBase of Enrichr. Following intersection, a TF-mRNA-miRNA regulatory network was established and visualized by Cytoscape. Python package “scenic” was used to identify specific TF of each cell cluster to validate the network. We generated transcription factor activities using default parameters and identified possible TF by a threshold of 10.

### Statistical analysis

Between-group comparison was performed using the Wilcoxon test. Correlation analysis was completed with the Spearman method and the result was displayed in a network. Without special statement, *p* < 0.05 was considered as statistically significant.

## Results

### scRNA-seq profile of chondrocytes in human osteoarthritis

**FIGURE 1:**
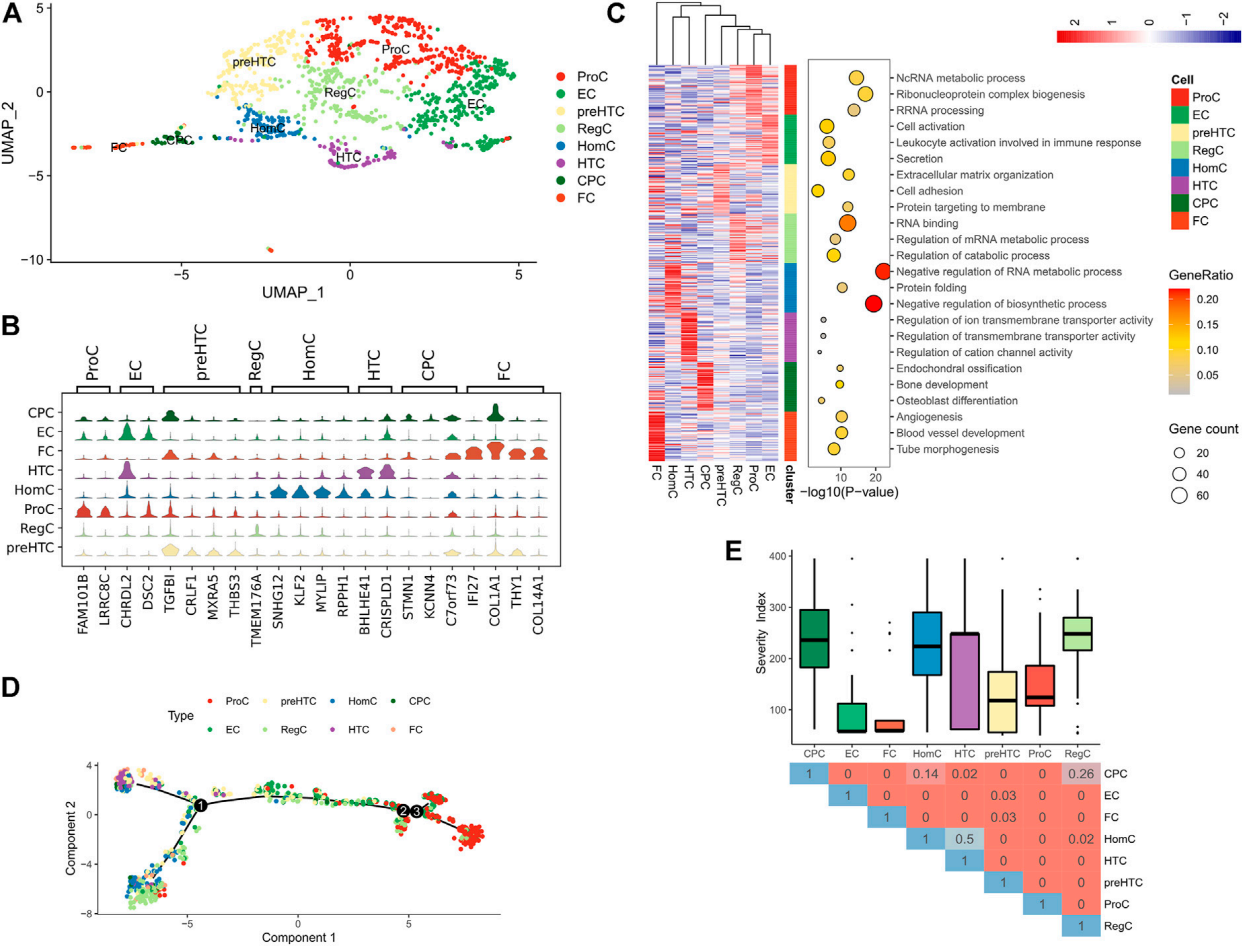
Sub-classification of chondrocytes in OA tissues. **(A)** UMAP plot of all chondrocytes, which are distinguished by color; **(B)** Violin plots showing the expression of canonical marker genes of each chondrocyte type; **(C)** The Heatmap (left) of the expression of the top 50 differential genes in each cell cluster (Z-score normalized) and the representative GO terms (right); **(D)** Differentiation trajectory of each cell cluster (distinguished by color) in OA tissue; **(E)** The Box plots showing the Severity score of the 8 cell clusters (upper) and the Heatmap showing the difference represented by p value in Wilcoxon test (below).

scRNA-seq was performed in the GSE104782 dataset. Following quality control for the transcriptome data of the total 1,600 chondrocytes (500<=gene number<=5,000), 1,343 chondrocytes were obtained for further analysis (Supplementary Figure S1A). Uniform manifold approximation and projection (UMAP) was used to cluster the 1,343 chondrocytes into 8 clusters of known cell lineages, with 10 set as the appropriate number of principal components (PCs). According to the canonical gene markers, the 8 cell clusters were annotated as: cartilage progenitor cells (CPCs), effector chondrocytes (ECs), fibrocartilage chondrocytes (FCs), homeostatic chondrocytes (HomCs), hypertrophic chondrocytes (HTCs), prehypertrophic chondrocytes (preHTCs), proliferative chondrocytes (ProCs) and regulatory chondrocytes (RegCs) (Figures 1A,B, Supplementary Table S1). Further functional annotation for the gene markers revealed that ECs were actively secreted and associated with cell activation and lymphocyte activation (Supplementary Table S2); FCs were involved in angiogenesis and blood vessel development (Figure 1C) and highly expressed fibroblast markers COL1A1 and COL14A1 (Figure 1B). Additionally, canonical gene markers of the 8 clusters were processed for Disease Ontology (DO) analysis, and most of them were in relation to bone diseases and inflammation (Supplementary Figure S2; Supplementary Table S3). Moreover, the pseudotime analysis as implemented by R package “monocle” revealed that FCs, HTCs and preHTCs were mainly located at the start point of the trajectory, while ProCs were majorly present at the end point. RegCs and ECs were distributed along the trajectory (Figure 1D). The results indicated that the 8 cell clusters were functionally different. FCs may participate in the initial fiber degeneration, while RegCs and ECs may play a regulatory role during the whole process of arthritis. Further analysis into the clinical significance of the 8 cell clusters was conducted with the Hospital for Special Surgery (HSS) scoring system and OA grading, the results of which demonstrated that ECs and FCs were associated with a higher disease severity than other cell clusters, suggesting their potential role in promoting progression of OA (Figure 1E). Therefore, we focused on ECs and FCs in subsequent analysis.

### Copy number variation in each cell cluster

**FIGURE 2:**
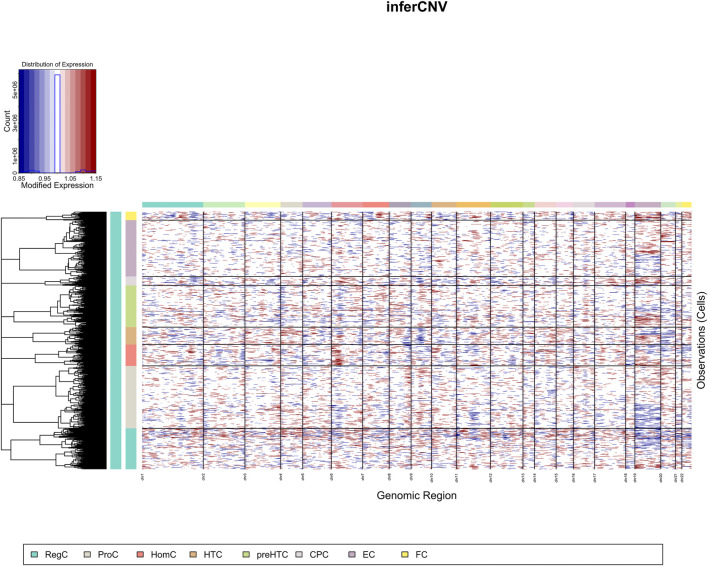
CNV landscape (the X-axis represents chromosome, and the Y-axis represents cell cluster).

CNV was analyzed in individual cell clusters using the package “inferCNV” and was found much more prevalent in ECs than in FCs (Figure 2).

### Effector chondrocytes and fibrocartilage chondrocytes exhibit strong immunogenicity

**FIGURE 3:**
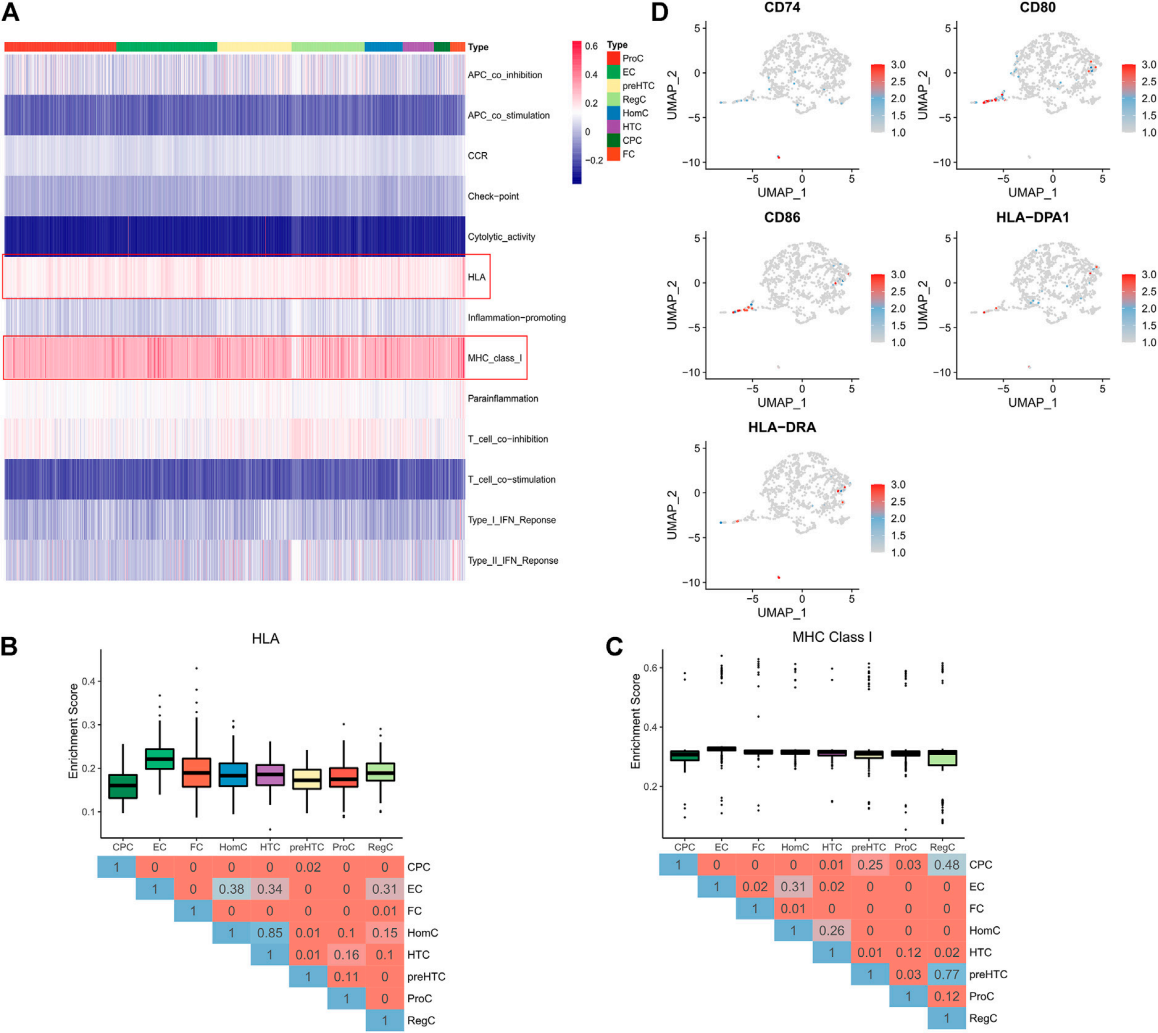
Immunogenicity of each cell cluster. **(A)** The Heatmap showing the activity of 13 immune pathways in cell clusters (pathways of interest are highlighted by red frames); **(B)** The Box plots showing the activity of the HLA pathway in 8 cell clusters (upper) and the Heatmap showing the difference represented by p value in Wilcoxon test (below); **(C)** The Box plots showing the activity of the MHC ClassⅠpathway in 8 cell clusters (upper) and the Heatmap showing the difference represented by p value in Wilcoxon test (below); **(D)** UMAP plots of each cell clusters colored by expression of marker genes.

Single sample GSEA (GSEA) algorithm was adopted to assess immune activity in each cell clusters, and the enrichment of 13 immune-related pathways was displayed in a Heatmap (Figure 3A). It was found that ECs and FCs, especially ECs, had higher enrichment scores of the HLA and MHC class1 pathways than other cell clusters, demonstrating that ECs and FCs possessed stronger immunogenicity (Figures 3B,C). Moreover, the expression of MHC genes was examined. The result revealed that CD74, CD80, CD86, HLA-DPA1 and HLA-DRA exhibited high expression in a small number of ECs and FCs (Figure 3D). These findings indicated that ECs and FCs may have some functions as immune cells during the progression of OA.

### Interactions of effector chondrocytes and fibrocartilage chondrocytes with other cell clusters

**FIGURE 4:**
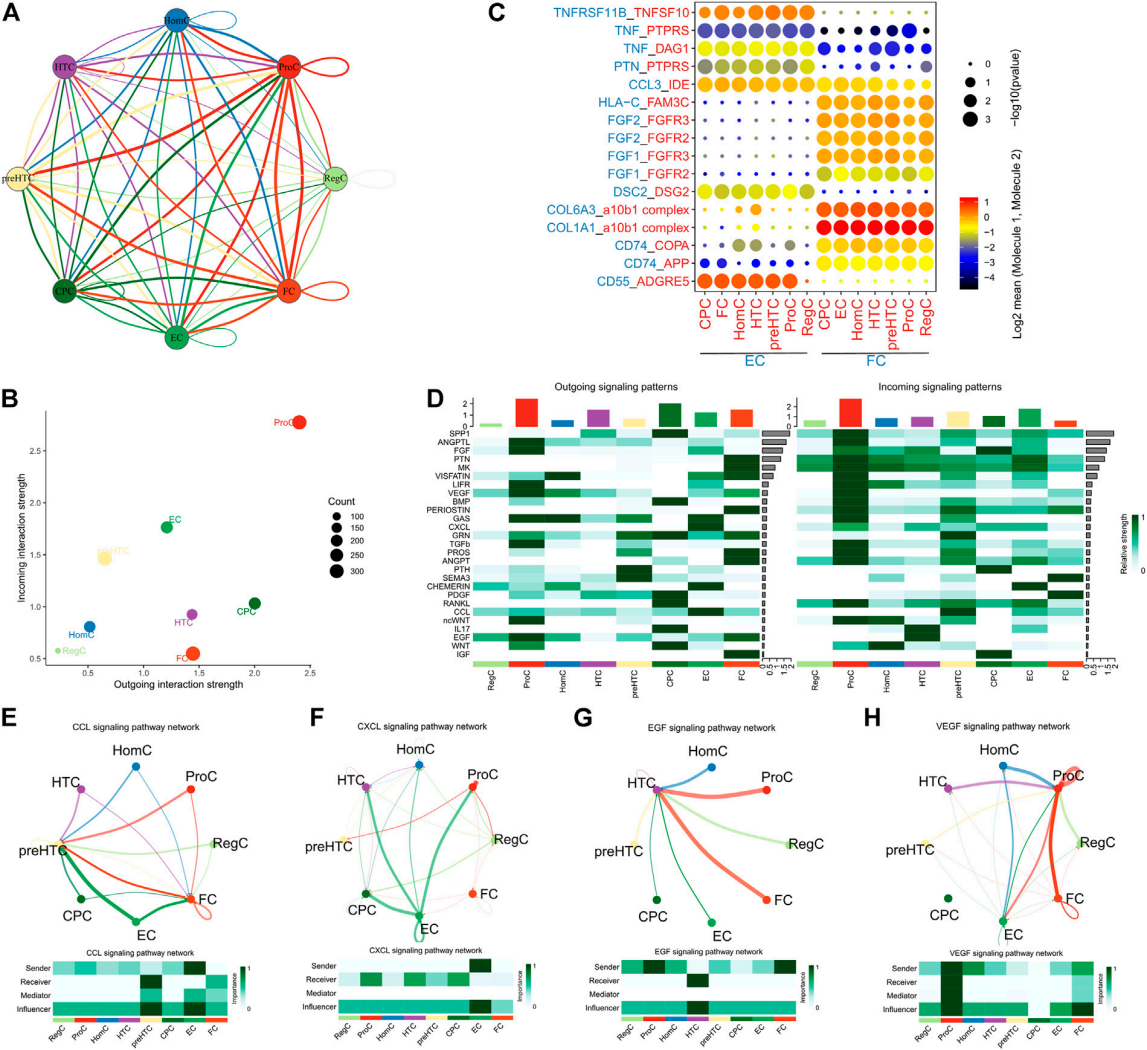
Cell-cell interactions. **(A)** The network showing the overall interactions between the 8 cell clusters (the thickness of the lines represents the number of receptor-ligand pairs); **(B)** The Scatter plot showing the number of interactions between the 8 cell clusters (the X-axis represents the number of outgoing interactions and the Y-axis represents the number of incoming interactions); **(C)** The point diagram showing the specific receptor-ligand interactions between ECs/FCs and other cell clusters (p value is represented by the circle size, and the mean expression of genes in ECs and FCs are represented by color); **(D)** The Heatmap showing the activity of signaling pathways involved in the interactions between the 8 cell clusters (left, outgoing signals; right, incoming signals); **(E)** ECs communicate with preHTCs and FCs through the CCL signaling pathway; **(F)** ECs communicate with CPCs, HTCs, HomCs and ProCs through the CXCL signaling pathway; **(G)** FCs communicate with HTCs through the EGF signaling pathway; **(H)** FCs communicate with ProCs through the VEGF signaling pathway.

Ligand-receptor interactions among the 8 cell clusters were explored using the CellPhoneDB. Extensive cell-cell communications were demonstrated among the 8 cell clusters (Figure 4A) and the detailed ligand-receptor interactions were revealed in Supplementary Table S4. We noted that ProCs had the most extensive interactions with other cell groups, followed by ECs (Figure 4B). Further analysis found that ECs and FCs highly expressed HLA-C and communicated with other cell groups via receptor FAM3C (Figure 4C). The result implied that ECs and FCs exhibited much stronger immunogenicity than other cell groups and they were involved in the immunoregulation. Notably, ECs also highly expressed CD55 and chemokine CCL3 while less TNF, and corresponding receptors were widely present in each cell groups. Therefore, ECs may play a more vital role in the immunoregulation. In addition, FCs mainly expressed COL1A1, COL6A3, FGF1 and FGF2 and communicated with other cell types via corresponding receptors, suggesting their role in fibrillogenesis and protein secretion (Figure 4C). This is in agreement with our previous finding. Subsequently, the specific pathways involved in the cell-cell communications were explored (Figure 4D). It was found that ECs communicated with preHTCs and FCs mainly via the CCL signaling pathway (Figure 4E). Additionally, ECs could also communicate with CPCs, HTCs and ProCs through the CXCL signaling pathway (Figure 4F). FCs communicated with HTCs predominantly through the EGF signaling pathway (Figure 4G) and with ProCs, RegCs and ECs *via* the VEGF signaling pathway (Figure 4H). In all, the results collectively indicated that ECs mainly play an immunoregulatory role in the cartilage microenvironment while FCs are the main participants in fibrillogenesis.

### Fibrocartilage chondrocytes have stronger immunogenicity and effector chondrocytes have higher metabolic activity

**FIGURE 5:**
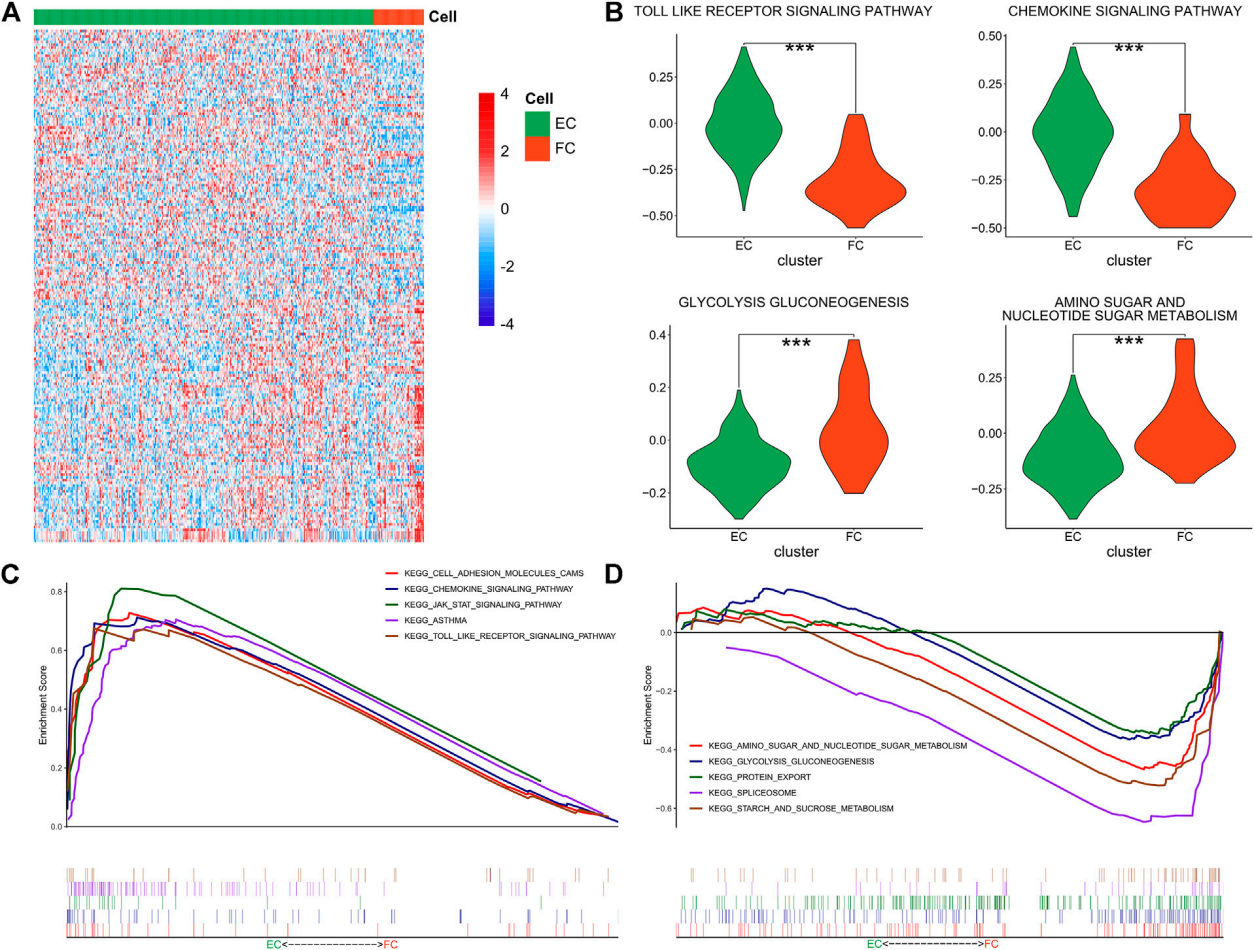
Functional enrichment of ECs and FCs. **(A)** The Heatmap showing the GSVA enrichment scores of KEGG pathways in ECs and FCs; **(B)** The Violin plots showing the specific GSVA results in ECs and FCs (***p < 0.001); **(C)** GSEA results in ECs; **(D)** GSEA results in FCs.

In the context of the difference in immunocompetence between FCs and ECs, the functional difference between the two cell groups was further investigated via GSVA and KEGG analyses. The enriched KEGG pathways of the two cell groups were displayed in the Heatmap (Figure 5A). It was found that ECs had higher activity in regulating chemokine signal transduction, Toll-like receptor and other signaling pathways, while FCs exhibited stronger metabolic activity involved in amino acid, ribose and glucose metabolisms (Figure 5B). GSEA revealed enhanced activity of Toll-like receptor, JAK/STAT and chemokine signaling pathways in ECs (Figure 5C) while augumented amino acid, ribose and glucose metabolisms in FCs, together with active glycolysis and gluconeogenesis (Figure 5D). The detailed results were shown in Supplementary Table S5. The above results illustrated that ECs have stronger immunogenicity than FCs and they participate in immunoregulation via regulating multiple signaling pathways. In the meantime, FCs show stronger catabolic function and have higher levels of amino acid metabolism and protein transportation involved in glycolysis and gluconeogenesis. All these findings are consistent with the previous report.

### Canonical gene markers of effector chondrocytes and fibrocartilage chondrocytes are predictors for the severity of Osteoarthritis

**FIGURE 6:**
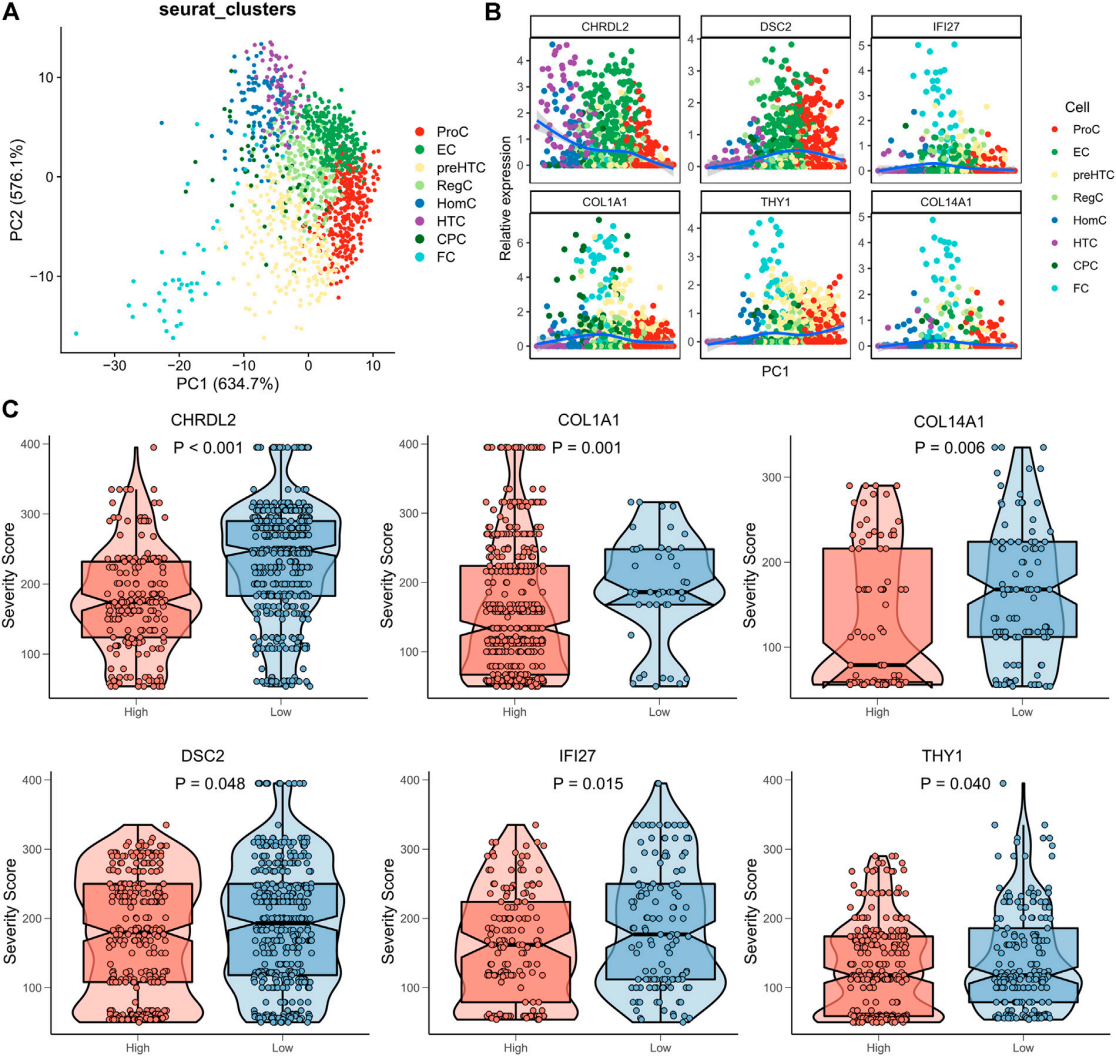
Clinical significance of marker genes of ECs and FCs. **(A)** PCA plots showing the distribution of the 8 cell clusters based on their marker genes; **(B)** Expression pattern of canonical marker genes along PC1; **(C)** The box plots showing the association between the Severity score and expression of gene markers (red for high expression and blue for low expression).

We had proved that ECs and FCs actively participated in the development of OA. Then, the clinical significance of their canonical gene markers (ECs: CHRDL2, DSC2; FCs: COL1A1, COL14A1, IFI27, THY1) was explored. PCA was firstly performed to confirm the strong correlation of ECs and FCs with their canonical gene markers. The results showed that the 8 cell clusters were distributed along PC1 (Figure 6A), and consistently, expression of the canonical gene markers of ECs and FCs also changed along PC1 (Figure 6B). Following exclusion of the samples not expressing the canonical markers, samples were sub-classified into the high- and low-expression groups according to the median expression of marker genes. We found that samples expressing more marker genes had a higher degree of disease severity (Figure 6C), suggesting that these marker genes of ECs and FCs may accurately predict the disease status of OA.

### Bulk RNA-seq analysis

**FIGURE 7:**
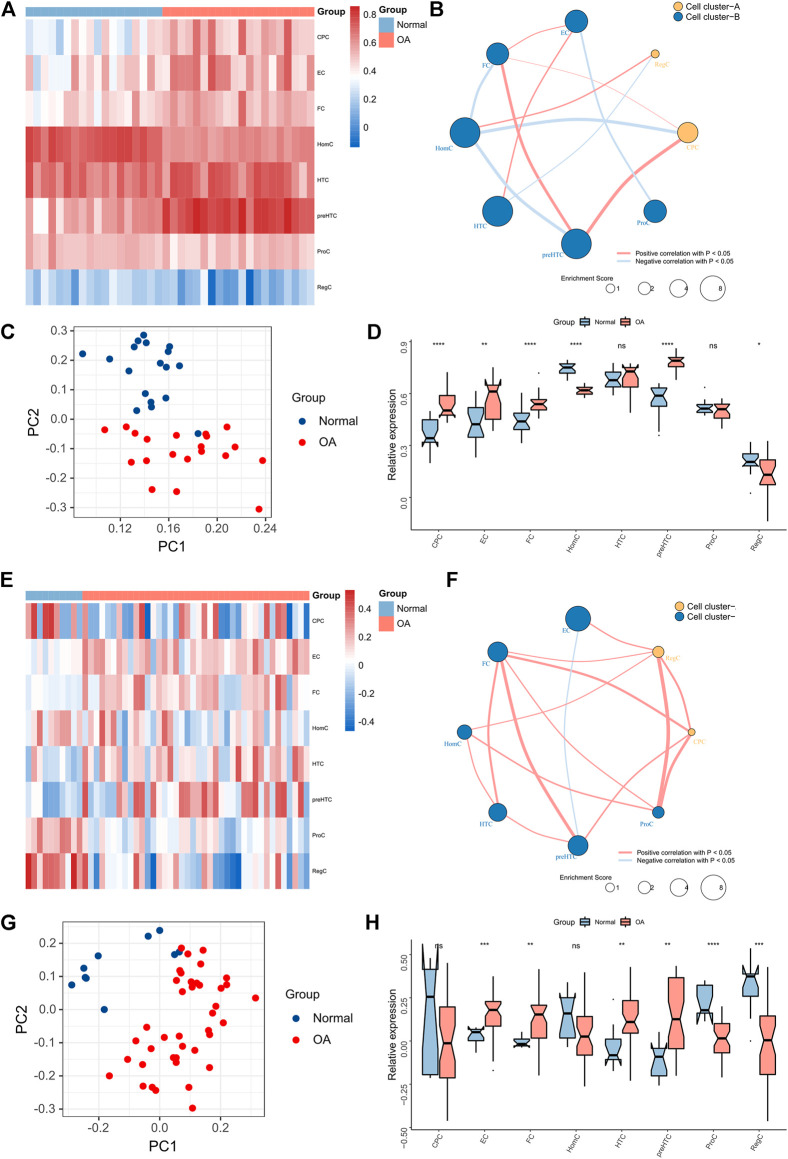
Chondrocytes profile in Bulk-seq data. **(A)** The Heatmap showing the relative abundance of the 8 cell clusters between OA and normal tissues in the GSE51588 dataset; **(B)** The network showing the significant associations between the 8 cell clusters in the GSE51588 dataset (the dot size represents the total enrichment score of chondrocytes and the clusters are distinguished by color; **(C)** PCA plot of chondrocytes based on enrichment scores in the GSE51588 dataset; **(D)** The box plots showing the relative abundance of the 8 cell clusters in OA and normal tissues in the GSE51588 dataset (*p < 0.05, **p < 0.01, ***p < 0.001; ns, not significant); **(E)** The Heatmap showing the relative abundance of the 8 cell clusters between OA and normal tissues in the GSE114007 dataset; **(F)** The network showing the significant associations between the 8 cell clusters in the GSE114007 dataset (the dot size represents the total enrichment score of chondrocytes and the clusters are distinguished by color; (G) PCA plot of chondrocytes based on enrichment scores in the GSE114007 dataset; **(H)** The box plots showing the relative abundance of the 8 cell clusters in OA and normal tissues in the GSE114007 dataset (*p < 0.05, **p < 0.01, ***p < 0.001; ns, not significant).

Bulk RNA-seq data from two independent datasets, GSE51588 and GSE114007, were used to validate the role of ECs and FCs in OA development. ssGSEA algorithm was firstly applied to estimate the enrichment score of each cell cluster in samples based on the gene markers (Figures 7A,E). Then, correlation analysis was conducted and showed that FCs were positively associated with CPCs and preHTCs, while ECs showed weak correlations with other cell clusters (Figures 7B,F). It is noteworthy that FCs were significantly associated with most cell groups in both GSE51588 and GSE114007, suggesting that FCs more actively communicate with other cells than ECs. Moreover, PCA demonstrated that the enrichment score of each cell cluster could well differentiate OA samples from healthy samples (Figures 7C,G). ECs and FCs had remarkably increased enrichment scores in OA samples than healthy samples (Figures 7D,H). All these results showed that the ECs and FCs may promote the development of OA and could be used as predictors for OA disease severity with high accuracy.

### Validation of the diagnostic performance of gene markers of effector chondrocytes and fibrocartilage chondrocytes in osteoarthritis

**FIGURE 8:**
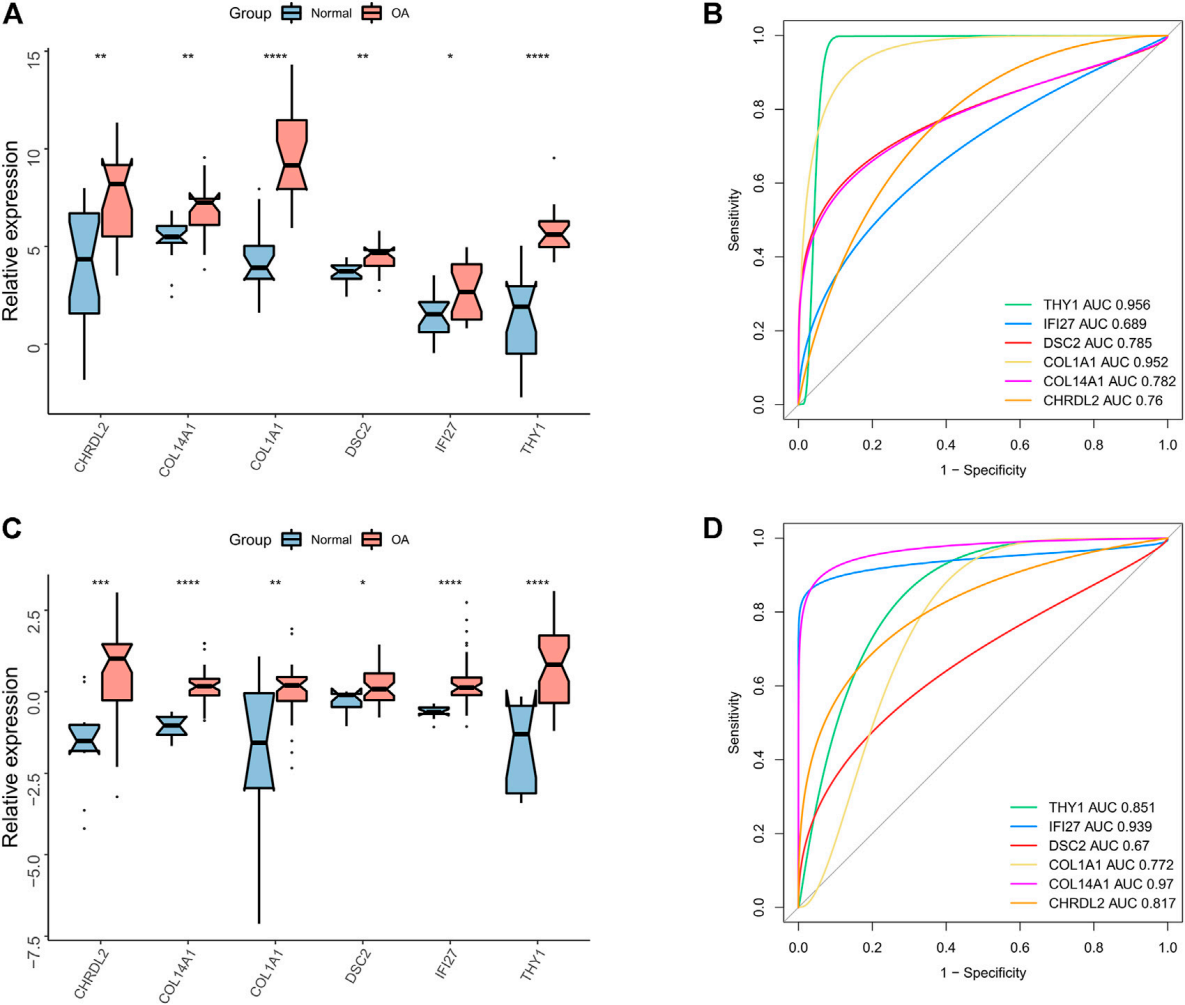
Diagnostic performance of canonical gene markers. **(A)** The box plots showing the expression difference between OA and normal tissues in expression of canonical gene markers in the GSE51888 dataset (*p < 0.05, **p < 0.01, ***p < 0.001; ns, not significant); **(B)** ROC curve of the gene markers in the GSE51888 dataset; **(C)** The box plots showing the expression difference between OA and normal tissues in expression of canonical gene markers in the GSE114007 dataset (*p < 0.05, **p < 0.01, ***p < 0.001; ns, not significant); **(D)** ROC curve of the gene markers in the GSE114007 dataset.

The diagnostic performance of the gene markers of ECs and FCs (CHRDL2, DSC2, COL1A1, COL14A1, IFI27, THY1) in OA was further analyzed in the GSE51588 and GSE114007. Significantly up-regulated expression of the gene markers was found in OA samples (Figures 8A,C). In addition, ROC curves showed that all the 6 gene markers could well predict OA in the GSE51588 and GSE114007 (all AUC>0.6) (Figures 8B,D).

### Transcription factor (FC) -mRNA-miRNA regulatory network

**FIGURE 9:**
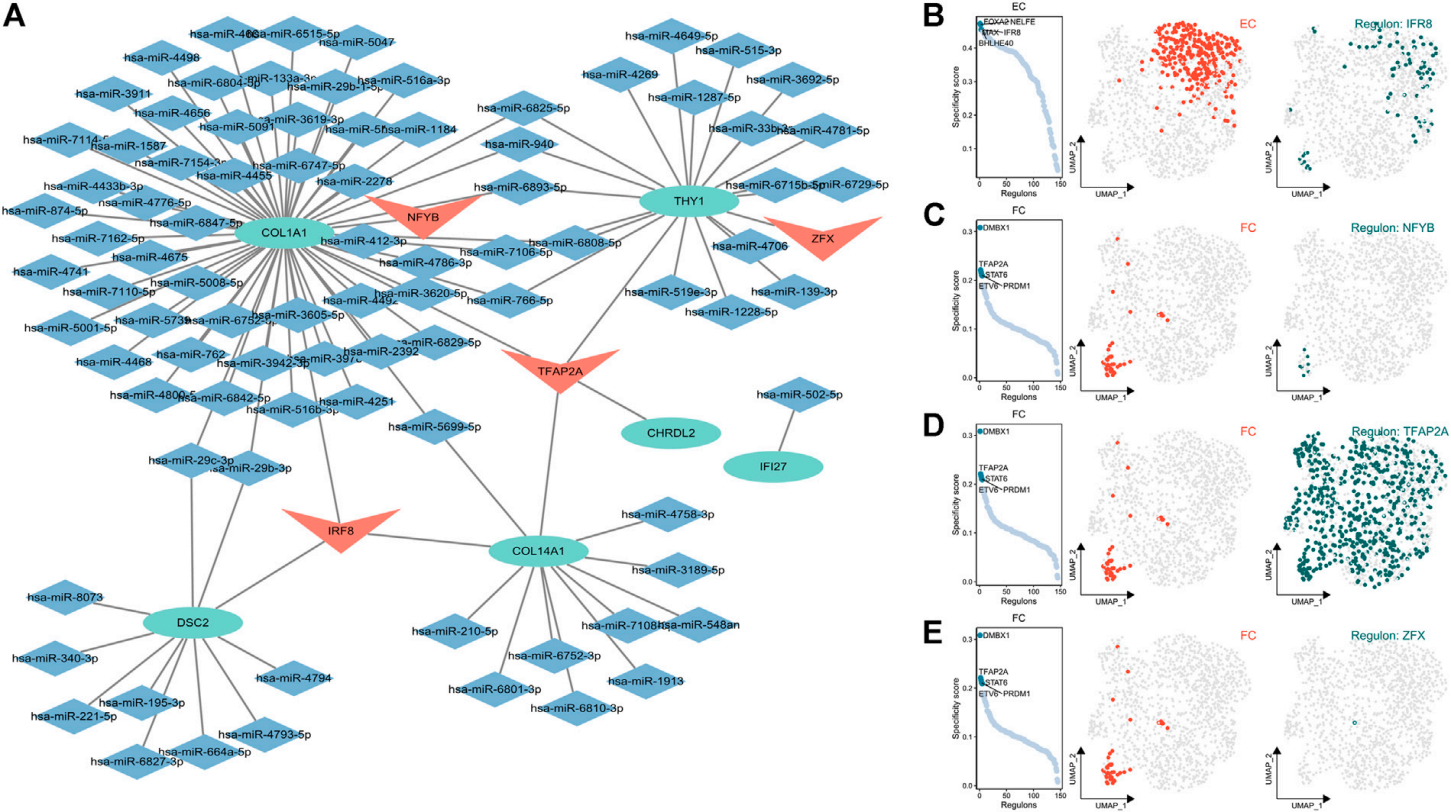
Establishment of TF-mRNA-miRNA regulatory network. **(A)** The TF-mRNA-miRNA regulatory network (TF, v-shaped frame; mRNA, oval shape; miRNA, rhombus); **(B)** Activity of TFs specific to ECs (from left to right: TF rank based on the regulon score, UMAP plot of the regulon activity, TF IFR8 regulon activity); **(C–E)** Activity of TFs specific to FCs (from left to right: TF rank based on the regulon score, UMAP plot of the regulon activity, TF **(C)** NFY8, (TFAP2A), and (ZFX) regulon activity).

A TF-mRNA-miRNA regulatory network of the gene markers of ECs and FCs (CHRDL2, DSC2, COL1A1, COL14A1, IFI27, THY1) was constructed to reveal the underlying mechanism by which the gene markers regulate OA progression. Upstream miRNA and TF of the gene markers were searched from the Enrichr database. Combining the miRNA-mRNA and TF-mRNA pairs, a TF-mRNA-miRNA regulatory network was correspondingly established, including 4 TFs, 87 miRNAs and 6 mRNAs (Figure 9A). Most of the gene markers were found to be regulated by both IRF8 and TFAP2A. Subsequently, TFs specific to ECs and FCs were inferred using the Scenic. IRF8 was specific to ECs and had higher transcriptional activity in ECs and FCs than in other cell clusters (Figure 9B). We also found that NFYB, TFAP2A and ZFX were specific to FCs and also exhibited stronger transcriptional activity in FCs than ECs (Figures 9C–E). Notably, TFAP2A had high transcriptional activity in almost all cell clusters (Figure 9D), suggesting its potential role in OA development.

## Conclusion

This study identified two subtypes of chondrocytes that advance the progression of OA, including ECs and FCs, with the former mainly responsible for cartilage degradation via affecting fiber degeneration while the latter for tissue inflammation via functions as immune cells. This finding provides new insight into the cellular heterogeneity and pathophysiology of OA. Moreover, their canonical gene markers, including CHRDL2, DSC2, COL1A1, COL14A1, IFI27, and THY1, can help for early diagnosis and precision treatment of OA.

## Discussion

OA is the most common age-associated chronic degenerative disease of articular cartilage and regarded as the leading cause of disability (Abusarah et al., 2017). Currently, arthroplasty is used as an effective approach to treat symptomatic end-stage OA, but it is limited in clinical application due to the dissatisfied outcome, limited longevity of prosthesis, and high economic burden (Glyn-Jones et al., 2015). Under the background of aging population and increasing economic burden, more attention has been paid to the early detection and treatment of OA (Schnitzer et al., 2015; Pan et al., 2016). The emergence of biomarkers helps for diagnosis of OA and formulation of new treatment strategies. However, markers specific to chondrocytes are limited given the heterogeneity of chondrocytes, which prompts us to identify the role of chondrocytes in pathogenesis of OA. In the present study, we used scRNA-seq technique to study the heterogeneity of chondrocytes in OA and revealed their immunogenicity and interactions among different cell types. In addition, we identified new biomarkers available to predict OA and validated their performance using the Bulk RNA-seq technique. Our findings are conducive to further studying the heterogeneity and biological functions of chondrocytes in OA and provide new targets for early diagnosis and treatment of OA.

Due to the highly complicated heterogeneity of chondrocytes in OA, we focused on the subtypes of chondrocytes that potentiate OA development. We found that ECs and FCs had a significant effect in promoting OA development. Functional analysis revealed that ECs exhibited stronger immunocompetence and secretion activity, while FCs had higher property of fibroblasts. All these results suggested that ECs may participate in the activation of immune cells and the synthesis of proteoglycan during OA progression, and FCs may be involved in fiber degeneration. Research found that CNV was abundant in MHC and TCR genes, which potentiated the ability of the immune system to recognize genetic variants, indicating that CNV might be an important mechanism that boosts immune diversity (Bailey et al., 2002; Nguyen et al., 2006; Olsson and Holmdahl, 2012). Similarly, our study found that ECs had a higher prevalence of CNV than FCs, suggesting their higher immune diversity and stronger chromosomal heterogeneity.

In general cases, OA is considered non-inflammatory but can manifest inflammatory phenotypes. Increasing studies has also noted that chronic low-grade inflammation can result in symptoms and disease progression (Sokolove and Lepus, 2013; Rhoads et al., 2017). Our study revealed that ECs and FCs had the highest activity of MHC and HLA pathways than other cell groups and highly expressed MHC genes. In the meantime, ECs and FCs actively communicated with other cell groups. These results demonstrated that FCs and ECs had stronger immunogenicity as compared to other cell types (Blais et al., 2011; Calis et al., 2013; Rossjohn et al., 2015). Interestingly, we found that ECs communicated with other cells mainly *via* CD55, which indicated that ECs potentiated OA development mainly through regulating the complement system of innate immunity (Hamann et al., 1999; Manferdini et al., 2016). Additionally, ECs had higher levels of CCL3 and TNF, showing their active participation in antigen-presentation and chemokine signaling pathway (Kalliolias and Ivashkiv, 2016; Jordan et al., 2018). Moreover, further analysis showed that ECs communicated with other cell types mainly via CCL and CXCL, which also indicated that ECs mediated the inflammation and immune cell recruitment in OA (Raghu et al., 2017; Ding et al., 2020). Furthermore, ECs exhibited stronger activity in regulating multiple signaling pathways including Toll-like receptor, JAK/STAT and chemokine signaling pathways. All these results implied that ECs are more potent in immunoregulation and advance inflammation and disease progression in OA, consistent with the previous report (Ji et al., 2019).

It is a fact that metabolic activities, including catabolism and anabolism, are essential to maintain the optimal cartilage function and promote cartilage tissue self-repair (Zhang et al., 2015). During the development of OA, glycolysis gradually becomes the main approach of metabolism, causing damages to the normal anabolic process of extracellular matrix (ECM) (Suurmond et al., 2015; Zhang et al., 2015; Hui et al., 2016). In the present study, FCs, compared to ECs, exhibited stronger metabolic activity in amino acid, ribose and glucose metabolisms and had active glycolysis and gluconeogenesis. In the meantime, they were found to actively communicate with other chondrocytes *via* the FGF family members, COL1A1 and COL6A3, showing a strong capability to secret fibers and their participation in cartilage tissue repair (Katoh, 2016; Xie et al., 2020). Moreover, the role of FCs in regulating fibrillogenesis was also proved by the participation of EGF and VEGF pathways in their interactions with other cell types (Schultz et al., 1991; Apte et al., 2019). It has been established that active glycolysis and gluconeogenesis may result in synthesis of abnormal proteoglycan, which then impedes the self-repair of cartilage tissue.

Collectively, our study demonstrated that ECs and FCs are important players during the progression of OA. More specifically, FCs mainly affect fiber degeneration and cartilage tissue repair to induce cartilage degradation, while ECs mainly play a role as immune cells via regulating inflammation and multiple signaling pathways, leading to joint tissue inflammation. In further steps, we also used Bulk RNA-seq data to validate the performance of ECs and FCs in predicting OA severity as well as the performance of their gene markers including CHRDL2, DSC2, COL1A1, COL14A1, IFI27, and THY1, which showed high expression in OA samples. Since the gene markers studied here are specific to OA, they can be used as flow cytometry markers with higher effectiveness than traditional markers and thus are highly valuable in early diagnosis and precision treatment of OA.

One of the limitations is the retrospective design of the study, and the sub-classification of chondrocytes requires further validation through *in vivo* and *ex vivo* experiments. In addition, the biological functions of ECs and FCs deserve further exploration. Furthermore, a larger-scale clinical cohort study is also on demand to investigate the performance of ECs, FCs and their canonical gene markers in early diagnosis of OA.

## Data Availability

The datasets presented in this study can be found in online repositories. The names of the repository/repositories and accession number(s) can be found in the article/Supplementary Material.
